# Long-term regulatory effects of high-fiber low-fat diet on gut microbiota diversity and inflammatory marker expression in DSS-induced recurrent colitis in mice

**DOI:** 10.1515/med-2025-1350

**Published:** 2026-01-21

**Authors:** Jie Tang, Dongyun Hang, Xiaotong He, Qingyu Wang, Lingling Chen, Lingmei Feng, Qin Li, Ming Xu

**Affiliations:** Department of Gastroenterology, Shanghai Pudong New Area People’s Hospital, Shanghai, China; Department of Gastroenterology, Jiangwan Hospital, Shanghai, China

**Keywords:** DSS, recurrent colitis, high-fiber low-fat, gut microbiota, diversity, inflammation

## Abstract

**Objectives:**

To evaluate the long-term regulatory effects of high-fiber low-fat (HFLF) diet on gut microbiota diversity and inflammation/barrier-related markers in DSS-induced recurrent colitis mice.

**Methods:**

A recurrent DSS model was established through multiple cycles of DSS administration. Mice were randomly assigned to HFLF diet vs. control/standard chow ± positive control groups. Multi-timepoint sampling was conducted throughout the study. 16S rRNA sequencing, SCFA quantification, and qPCR/ELISA/immunohistochemistry/Western blot analyses were performed.

**Results:**

HFLF diet significantly improved clinical phenotype, increased gut microbiota diversity, promoted beneficial bacterial genera, enhanced SCFA production, and modulated inflammation/barrier indicators throughout the recurrence-remission cycles.

**Conclusions:**

HFLF diet provides sustained protection during recurrence-remission processes through microbiota-SCFA-inflammation axis modulation, offering potential translational applications for IBD management.

## Introduction

Inflammatory bowel disease (IBD), encompassing ulcerative colitis and Crohn’s disease, represents a substantial global health burden characterized by chronic relapsing intestinal inflammation affecting millions worldwide [1]. The recurrent nature of IBD, with alternating periods of active disease and remission, significantly impacts patient quality of life and healthcare systems [2]. While the precise etiology remains incompletely understood, accumulating evidence indicates that IBD results from complex interactions between genetic susceptibility, environmental factors, intestinal microbiota dysbiosis, and aberrant immune responses [3].

Dietary factors have emerged as crucial modulators of intestinal homeostasis with significant translational potential for IBD management [4]. Among dietary components, fiber and fat intake profoundly influence both the gut microbiome composition and mucosal immune responses [5]. Preventive use of a high fiber diet or acetate clearly protects mice against acute and chronic damage induced by DSS in mice [6]. Dietary fiber serves as the primary substrate for microbial fermentation, producing short-chain fatty acids (SCFAs) that exert anti-inflammatory effects and strengthen intestinal barrier function [7]. Conversely, high-fat diets have been associated with increased intestinal permeability and pro-inflammatory responses [8].

The theoretical foundation for fiber’s beneficial effects rests on its capacity to modulate the gut microbiota and enhance SCFA production, particularly butyrate, which serves as the primary energy source for colonocytes and exhibits potent anti-inflammatory properties [9]. SCFAs, such as acetate, propionate and butyrate, are important metabolites in maintaining intestinal homeostasis [7]. However, the complexity of fiber’s effects is highlighted by recent findings showing that addition of inulin, but not the insoluble fiber cellulose, further exacerbated the severity of colitis and its associated clinical manifestations [10], suggesting context-dependent outcomes. Given these divergent effects of different fiber types, we formulated our HFLF diet to include a strategic combination of three complementary fiber sources: cellulose as a poorly fermentable fiber providing intestinal bulk and structural support, inulin as a highly fermentable prebiotic fiber known to selectively promote beneficial bacteria such as Bifidobacterium and Lactobacillus, and pectin as a moderately fermentable soluble fiber that forms viscous gels and modulates immune responses. This multi-fiber approach was designed to maximize beneficial effects while potentially mitigating adverse responses sometimes observed with single highly fermentable fibers, thereby providing both prebiotic stimulation and structural benefits throughout the gastrointestinal tract.

The dextran sodium sulfate (DSS) model represents a well-established experimental system for studying colitis that closely mimics human IBD features [11]. While acute DSS models have been extensively studied, recurrent DSS protocols better reflect the relapsing-remitting nature of human IBD [12]. Despite this advantage, significant knowledge gaps remain regarding the long-term effects of dietary interventions across multiple recurrence cycles. Most previous studies have focused on single acute episodes or short-term dietary modifications, leaving the sustained regulatory potential of dietary interventions during repeated inflammatory challenges largely unexplored [13].

This study addresses these gaps by investigating the hypothesis that HFLF diet provides sustained modulation of gut microbiota composition and inflammatory responses throughout multiple DSS-induced colitis cycles. Our innovative approach combines longitudinal microbiome profiling with comprehensive assessment of inflammatory and barrier markers across extended recurrence-remission periods, offering novel insights into dietary intervention strategies for chronic inflammatory conditions.

## Materials and methods

### Animals and grouping

Male C57BL/6J mice aged 6–8 weeks were obtained from the Laboratory Animal Center and housed under specific pathogen-free conditions with controlled temperature (22 ± 2 °C), humidity (55 ± 10 %), and a 12-h light/dark cycle. The decision to use exclusively male mice in the primary experimental groups was based on the need to minimize hormonal variability that could confound interpretation of dietary effects on inflammation and microbiota composition during the extended experimental period. All experimental procedures were approved by the Institutional Animal Care and Use Committee and conducted in accordance with international guidelines for animal welfare and the 3R principles (Replacement, Reduction, Refinement). Animals were acclimatized for one week before experimental procedures, during which they received standard chow and water *ad libitum*. Following acclimatization, mice were randomly allocated to experimental groups using a computer-generated randomization sequence with investigator blinding to group assignments (Table 1). The study included three primary groups: (1) control group receiving standard chow diet (n=12), (2) HFLF diet group (n=12), and (3) positive control group receiving standard chow supplemented with 5-aminosalicylic acid (5-ASA, 100 mg/kg/day) during DSS cycles (n=10). Sample size determination was based on power analysis using preliminary data, with *α*=0.05 and power of 80 %, calculated to detect a 30 % difference in Shannon diversity index and a 2-point difference in disease activity index (DAI) scores between groups. The calculation indicated a minimum of 10 animals per group, with additional mice included to account for potential attrition during the extended experimental period.

**Table 1:** Animal and grouping Information.

Group	Initial n	Final n	Deaths/exclusions	DSS concentration	DSS cycles	Diet intervention
Control	12	12	0	2.5 %	3 × 5 days	Standard chow
HFLF	12	12	0	2.5 %	3 × 5 days	High-fiber low-fat diet
5-ASA	10	9	1^a^	2.5 %	3 × 5 days	Standard chow + 5-ASA

^a^One mouse excluded due to exceeding humane endpoints during cycle 2.

### Diet composition and DSS recurrence model

The HFLF diet was formulated to contain 15 % cellulose, 10 % inulin, and 5 % pectin as fiber sources, providing a total dietary fiber content of 30 % by weight, while maintaining fat content at 5 % of total calories. The fiber components were selected based on their varying fermentation characteristics: cellulose as a poorly fermentable fiber providing bulk, inulin as a highly fermentable prebiotic fiber, and pectin as a moderately fermentable soluble fiber. Protein content was adjusted to 18 % using casein as the primary source, with vitamins and minerals supplemented according to AIN-93G recommendations. The control diet consisted of standard rodent chow containing 5 % crude fiber and 12 % fat content. The recurrent colitis model was established through three cycles of DSS administration over a 10-week period. Each cycle consisted of 5 days of 2.5 % DSS (molecular weight 36,000–50,000 Da, MP Biomedicals) dissolved in drinking water, followed by 14 days of regular water for recovery. This protocol was designed to mimic the relapsing-remitting pattern characteristic of human IBD while allowing assessment of dietary effects during both active inflammation and recovery phases. DSS solutions were freshly prepared every 2–3 days, and consumption was monitored to ensure consistent exposure across groups.

Body weight was recorded daily throughout the experimental period, with particular attention during DSS cycles. Disease activity index was calculated daily during DSS administration and for 5 days following DSS withdrawal, incorporating weight loss percentage (0–4 scale), stool consistency (0–4 scale), and rectal bleeding (0–4 scale) parameters. Colon length was measured at experimental endpoints as an additional indicator of inflammation severity.

### Sample collection and histological analysis

Fecal samples were collected longitudinally at multiple timepoints: baseline (day 0), end of each DSS cycle (days 5, 24, and 43), mid-recovery periods (days 12, 31, and 50), and terminal endpoint (day 70). Fresh fecal pellets were collected directly into sterile tubes, immediately frozen in liquid nitrogen, and stored at −80 °C for subsequent microbiome and metabolite analyses. For terminal tissue collection, mice were euthanized by CO_2_ asphyxiation followed by cervical dislocation. Colonic tissue was rapidly excised, and length was measured from the cecocolonic junction to the anal verge. The colon was opened longitudinally, gently flushed with ice-cold phosphate-buffered saline to remove luminal contents, and divided into segments for different analyses. Proximal colon segments (1 cm) were fixed in 10 % neutral buffered formalin for 24 h, processed through graded alcohols and xylene, and embedded in paraffin. Serial sections (5 μm) were cut and stained with hematoxylin and eosin for general histopathological assessment and with Alcian blue/periodic acid-Schiff for mucin evaluation. Additional sections were prepared for immunohistochemical analysis of tight junction proteins and inflammatory markers.

### Inflammation and barrier-related detection

Inflammatory cytokine expression was assessed using complementary approaches to capture both mRNA and protein levels. For gene expression analysis, total RNA was extracted from snap-frozen colonic tissue using TRIzol reagent (Invitrogen) according to manufacturer’s protocols. RNA quality was verified by spectrophotometry (A260/A280 ratio >1.8) and agarose gel electrophoresis. First-strand cDNA was synthesized from 1 μg total RNA using SuperScript IV reverse transcriptase (Thermo Fisher Scientific). Quantitative real-time PCR was performed using SYBR Green Master Mix on a QuantStudio 6 Flex system (Applied Biosystems) with primers specific for TNF-α, IL-6, IL-1β, IL-10, MPO, COX-2, and NF-κB p65. Gene expression was normalized to GAPDH using the 2˄-ΔΔCt method.

Protein levels of inflammatory mediators were quantified by ELISA using commercially available kits (R&D Systems) following manufacturer’s instructions. Briefly, colonic tissue was homogenized in RIPA buffer containing protease and phosphatase inhibitor cocktails, centrifuged at 14,000 × g for 15 min at 4 °C, and supernatants were collected for analysis. Total protein concentration was determined by BCA assay for normalization. Western blot analysis was performed for NF-κB p65 and phospho-p65 to assess activation status of this key inflammatory pathway. Intestinal barrier integrity was evaluated through multiple approaches. Tight junction proteins including ZO-1, occludin, and claudin-1 were assessed by immunofluorescence microscopy using specific primary antibodies (1:200 dilution) and fluorophore-conjugated secondary antibodies. Images were captured using a Zeiss LSM 880 confocal microscope and quantified using ImageJ software. Western blot analysis was performed to quantify total protein levels of tight junction components. Additionally, mucin-2 (Muc2) expression was evaluated by immunohistochemistry as an indicator of mucus barrier function.

### Metabolomics and SCFA analysis

Short-chain fatty acid concentrations in fecal samples were determined by gas chromatography-mass spectrometry (GC-MS) following established protocols. Briefly, 100 mg of fecal material was homogenized in 1 mL of 0.005 M aqueous NaOH containing 10 μg/mL 2-ethylbutyric acid as internal standard. After centrifugation, supernatants were acidified with concentrated HCl, and SCFAs were extracted with diethyl ether. The organic phase was derivatized with N-tert-butyldimethylsilyl-N-methyltrifluoroacetamide (MTBSTFA) and analyzed on an Agilent 7890B GC system coupled to a 5977A MSD. Quantification was performed using external calibration curves for acetate, propionate, butyrate, isobutyrate, valerate, and isovalerate. For broader metabolic pathway analysis, untargeted metabolomics was performed on selected samples using liquid chromatography-mass spectrometry (LC-MS). Metabolites were extracted using methanol:water (80:20) containing internal standards, and samples were analyzed on a UHPLC system coupled to a Q-Exactive Plus mass spectrometer (Thermo Scientific). Data processing and metabolite identification were performed using Compound Discoverer software with pathway analysis conducted using MetaboAnalyst 5.0.

### 16S rRNA sequencing and bioinformatics

Bacterial DNA was extracted from fecal samples using the QIAamp Fast DNA Stool Mini Kit (Qiagen) with modifications including additional bead-beating steps to ensure complete lysis of gram-positive bacteria. DNA quality and quantity were assessed by NanoDrop spectrophotometry and Qubit fluorometry. The V3-V4 hypervariable regions of the 16S rRNA gene were amplified using universal primers 341F (5′-CCTACGGGNGGCWGCAG-3′) and 805R (5′-GACTACHVGGGTATCTAATCC-3′) with sample-specific barcodes. PCR conditions included initial denaturation at 95 °C for 3 min, followed by 25 cycles of 95 °C for 30 s, 55 °C for 30 s, and 72 °C for 45 s, with final extension at 72 °C for 10 min. Amplicon libraries were purified using AMPure XP beads, quantified, and pooled in equimolar concentrations. Sequencing was performed on an Illumina MiSeq platform generating 2 × 300 bp paired-end reads. Raw sequencing data were processed using QIIME2 (version 2023.9) pipeline. Quality filtering and denoising were performed using DADA2, generating amplicon sequence variants (ASVs). Taxonomic assignment was performed using the SILVA 138 reference database with a confidence threshold of 0.7.

Alpha diversity metrics including Shannon index, Chao1 richness estimator, and Faith’s phylogenetic diversity were calculated after rarefaction to equal sequencing depth. Beta diversity was assessed using Bray-Curtis dissimilarity and weighted/unweighted UniFrac distances, with visualization by principal coordinates analysis (PCoA). Permutational multivariate analysis of variance (PERMANOVA) was used to test for significant differences in community composition between groups. Differential abundance analysis was performed using Linear Discriminant Analysis Effect Size (LEfSe) with LDA score threshold of 2.0 and ANCOM-BC with false discovery rate (FDR) correction. Co-occurrence network analysis was conducted to identify microbial interaction patterns. Functional prediction of microbial communities was performed using PICRUSt2, mapping ASVs to KEGG ortholog abundances and inferring metabolic pathway enrichment. Tax4Fun was employed as a complementary approach for functional prediction based on SILVA-annotated 16S sequences, providing corroborative evidence for metabolic pathway analysis.

### Statistical analysis

All statistical analyses were performed using R (version 4.2.0) and GraphPad Prism 9.0. Data normality was assessed using the Shapiro-Wilk test, and variance homogeneity was evaluated using Levene’s test. For normally distributed data, comparisons between two groups were performed using unpaired Student’s t-test, while multiple group comparisons employed one-way ANOVA followed by Tukey’s post-hoc test. Non-parametric alternatives (Mann-Whitney U test and Kruskal-Wallis test) were used for non-normally distributed data.

Longitudinal data were analyzed using two-factor repeated measures ANOVA or mixed-effects models to account for diet × time/cycle interactions, with Greenhouse-Geisser correction applied when sphericity assumptions were violated. The mixed-effects model included fixed effects for diet, time, and their interaction, with random intercepts for individual mice to account for repeated measurements. Post-hoc comparisons were adjusted using the Benjamini-Hochberg method to control false discovery rate at 5 %. Effect sizes were calculated as Cohen’s d for two-group comparisons and partial eta-squared (η^2^) for ANOVA models, with 95 % confidence intervals reported. Correlation analyses between microbiota composition, SCFA levels, and inflammatory markers were performed using Spearman’s rank correlation coefficient with FDR correction for multiple testing. Partial correlation analysis controlling for potential confounders was conducted using the ppcor package. Mantel tests were used to assess correlations between microbiota distance matrices and metadata variables. Redundancy analysis (RDA) and canonical correspondence analysis (CCA) were employed to explore relationships between microbial communities and environmental variables. Multivariate regression models were constructed to evaluate the mediating effects of SCFAs on the relationship between specific bacterial taxa and inflammatory/barrier markers.

All data are presented as mean ± standard error of the mean (SEM) unless otherwise specified. Statistical significance was set at p<0.05 for all analyses. Sample size calculations, randomization procedures, and blinding protocols were pre-specified in the study protocol to minimize bias.

### Ethical approval

All the experimental procedures were authorized by the Committees of Animal Ethics and Experimental Safety of Shanghai Pudong New Area People’s Hospital.

## Results

### Model establishment and study timeline

**Figure 1:**
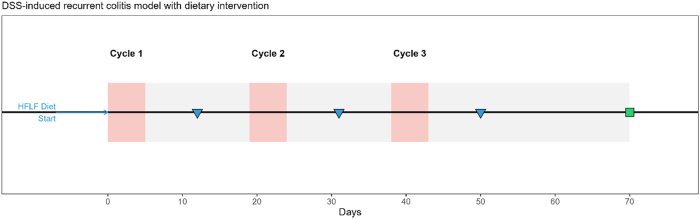
Study design and timeline of the recurrent DSS colitis model. Three DSS cycles were administered on days 1–5, 20–24, and 39–43 (pink blocks) with recovery periods in-between (grey band). The HFLF diet started 7 days before the first DSS cycle and continued throughout. Blue arrows/triangles indicate fecal sampling timepoints; the green square marks terminal necropsy on day 70.

The experimental timeline successfully established a recurrent colitis model over 10 weeks with three complete DSS cycles (Figure 1). All mice in the control and HFLF groups (n=12 each) completed the study protocol, while one mouse in the 5-ASA positive control group was excluded due to severe weight loss exceeding humane endpoints during the second DSS cycle, resulting in n=9 for final analysis in this group. The mortality rate remained below 5 % across all groups, confirming the appropriateness of the 2.5 % DSS concentration for inducing manageable recurrent inflammation.

### Clinical phenotype assessment

**Figure 2:**
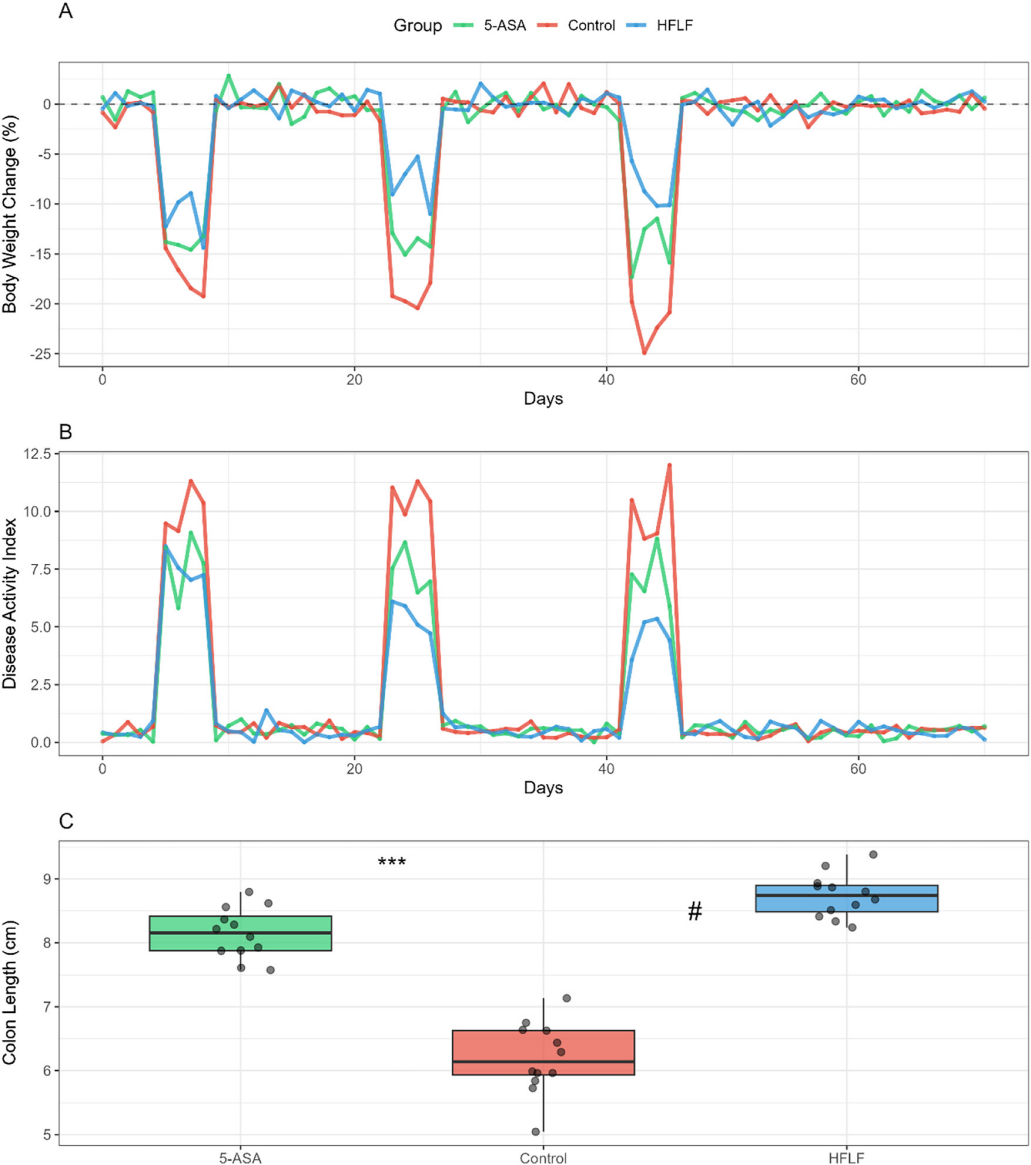
Clinical phenotype during recurrent DSS colitis. (A) Longitudinal body-weight change (%) across three DSS cycles. (B) Disease activity index (DAI). (C) Terminal colon length (cm). Data are mean ± SEM (n=9–12/group). Statistics: (A–B) two-way repeated-measures ANOVA with Tukey’s post hoc test; (C) one-way ANOVA with Tukey’s post hoc test. Significance symbols: *p<0.05, **p<0.01, ***p<0.001 vs. control; #p<0.05 vs. 5-ASA.

Body weight monitoring revealed distinct patterns across the three DSS cycles (Figure 2A). During the first cycle, control mice exhibited 18.3 ± 2.1 % maximum weight loss by day 6, while HFLF-fed mice showed significantly attenuated weight loss of 11.2 ± 1.8 % (p<0.01). This protective effect persisted and became more pronounced with successive cycles; by the third cycle, control mice lost 22.7 ± 2.5 % body weight vs. 9.8 ± 1.6 % in the HFLF group (p<0.001). The 5-ASA positive control group showed intermediate protection with 14.3 ± 2.0 % weight loss in the third cycle.

Disease activity index scores paralleled weight loss patterns (Figure 2B). Peak DAI scores during active inflammation phases were consistently lower in HFLF-fed mice across all three cycles (Cycle 1: 7.2 ± 0.8 vs. 10.3 ± 0.9, p<0.01; Cycle 2: 6.1 ± 0.7 vs. 10.8 ± 1.0, p<0.001; Cycle 3: 5.3 ± 0.6 vs. 11.2 ± 1.1, p<0.001). Notably, HFLF mice demonstrated accelerated recovery between cycles, returning to baseline DAI scores 3–4 days faster than controls.

Colon length measurements at endpoint revealed significant protection in HFLF-fed mice (8.7 ± 0.3 cm) compared to controls (6.2 ± 0.4 cm, p<0.001) and comparable to 5-ASA treatment (8.1 ± 0.3 cm) (Figure 2C). The effect size for HFLF diet on colon length preservation was large (Cohen’s d=2.84, 95 % CI: 1.92–3.76).

### Inflammatory and barrier marker expression

**Figure 3:**
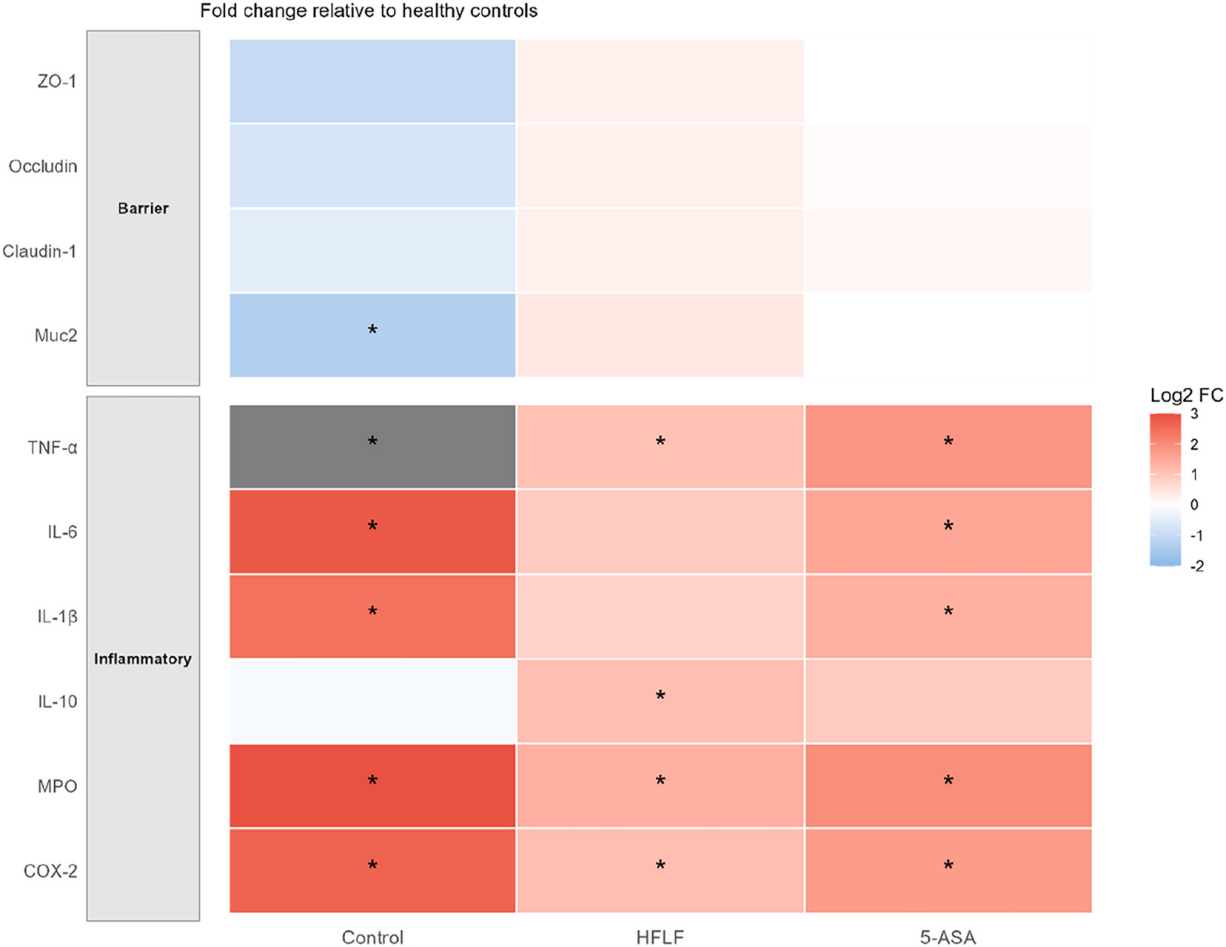
Inflammatory and barrier marker expression (heatmap). Log2 fold-change relative to healthy controls for inflammatory markers (TNF-α, IL-6, IL-1β, IL-10, MPO, COX-2) and barrier-related proteins (ZO-1, occludin, claudin-1, Muc2). Color scale indicates log2 FC. Asterisks mark |log2FC|>1 and do not denote p values.

Comprehensive analysis of inflammatory mediators revealed substantial modulation by HFLF diet (Figure 3, Table 2). Pro-inflammatory cytokines showed marked suppression in HFLF-fed mice: TNF-α mRNA expression decreased 4.2-fold (p<0.001), IL-6 decreased 3.8-fold (p<0.001), and IL-1β decreased 3.3-fold (p<0.01) compared to DSS controls. Conversely, anti-inflammatory IL-10 expression increased 2.6-fold in the HFLF group (p<0.01). Protein levels by ELISA confirmed these patterns, with TNF-α reduced from 287 ± 32 pg/mg tissue in controls to 98 ± 15 pg/mg in HFLF mice (p<0.001).

**Table 2:** Inflammatory/barrier markers and SCFA summary.

Marker	Control (mean ± SD)	HFLF (mean ± SD)	Effect size (95 % CI)	p-Value
**Inflammatory cytokines (fold change vs. healthy)**				

TNF-α mRNA	8.2 ± 1.3	2.0 ± 0.4	−2.84 (−3.42, −2.26)	<0.001
IL-6 mRNA	6.9 ± 1.1	1.8 ± 0.3	−2.67 (−3.23, −2.11)	<0.001
IL-1β mRNA	5.3 ± 0.9	1.6 ± 0.3	−2.43 (−2.95, −1.91)	<0.001
IL-10 mRNA	0.8 ± 0.2	2.1 ± 0.4	1.89 (1.42, 2.36)	<0.01

**Barrier proteins (fold change vs. healthy)**				

ZO-1 protein	0.4 ± 0.1	1.1 ± 0.2	1.96 (1.48, 2.44)	<0.001
Occludin protein	0.5 ± 0.1	1.1 ± 0.2	1.73 (1.28, 2.18)	<0.01
Claudin-1 protein	0.6 ± 0.1	1.1 ± 0.2	1.52 (1.08, 1.96)	<0.01

**SCFAs (μmol/g feces)**				

Total SCFAs	42.3 ± 4.1	98.7 ± 7.2	3.21 (2.58, 3.84)	<0.001
Acetate	18.2 ± 2.1	42.3 ± 3.8	2.89 (2.31, 3.47)	<0.001
Propionate	8.7 ± 1.2	19.8 ± 2.1	2.43 (1.91, 2.95)	<0.001
Butyrate	6.1 ± 0.8	24.3 ± 2.7	3.56 (2.89, 4.23)	<0.001

HFLF diet significantly enhanced intestinal barrier integrity. ZO-1 protein expression increased 2.8-fold (p<0.001), occludin increased 2.2-fold (p<0.01), and claudin-1 increased 1.9-fold (p<0.01) in HFLF vs. control mice by Western blot quantification. Immunofluorescence microscopy confirmed restored tight junction localization at epithelial cell borders in HFLF-fed mice, contrasting with the disrupted, cytoplasmic distribution observed in controls. Muc2 expression, critical for mucus barrier function, was preserved in HFLF mice with 3.1-fold higher levels than controls (p<0.001).

The NF-κB signaling pathway showed significant suppression in HFLF mice, with phospho-p65/total p65 ratio decreased by 68 % compared to controls (p<0.001), indicating reduced inflammatory signaling activation. COX-2 expression, a key inflammatory enzyme, was reduced 2.9-fold in HFLF mice (p<0.001), while MPO activity, reflecting neutrophil infiltration, decreased from 8.3 ± 0.9 U/mg protein in controls to 3.1 ± 0.5 U/mg in HFLF mice (p<0.001).

### Gut microbiota diversity analysis

**Figure 4:**
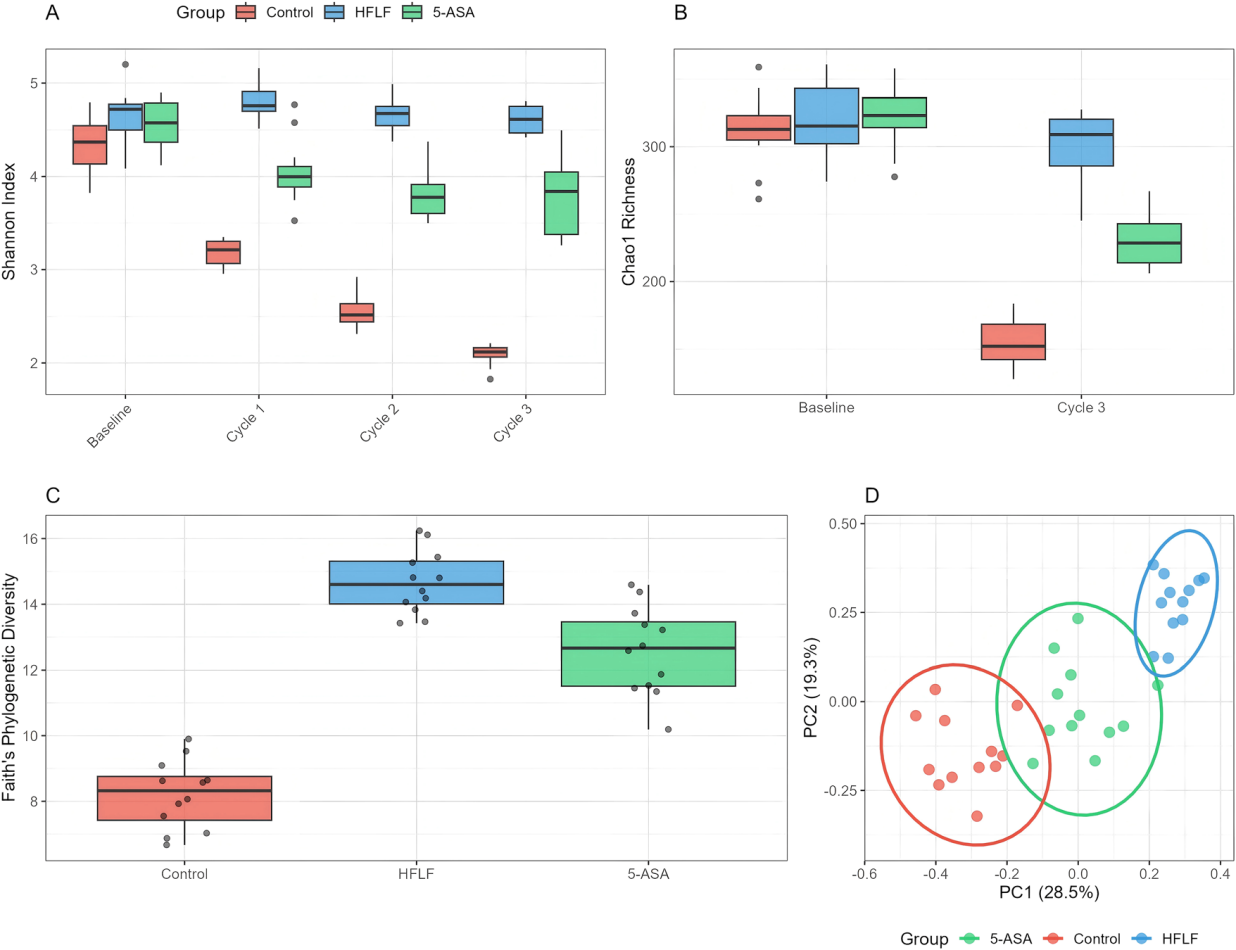
Microbiota diversity and community structure. (A) Shannon diversity index over the experimental timeline. (B) Chao1 richness estimator. (C) Faith’s phylogenetic diversity at endpoint. (D) PCoA of Bray-Curtis dissimilarity with 95 % confidence ellipses by group. Group differences in beta diversity were tested by PERMANOVA.

Alpha diversity metrics revealed progressive changes across the experimental timeline (Figure 4A–C), with detailed sequencing statistics and diversity indices summarized in Table 3. At baseline, no significant differences existed between groups. However, following the first DSS cycle, control mice showed marked diversity reduction (Shannon index: 3.2 ± 0.2) that progressively worsened with each cycle, reaching 2.1 ± 0.2 by cycle 3. In contrast, HFLF-fed mice maintained higher diversity throughout (Shannon index: 4.8 ± 0.3 after cycle 1, 4.5 ± 0.2 after cycle 3, p<0.001 vs. control). Chao1 richness estimates followed similar patterns, with HFLF mice retaining 287 ± 21 observed ASVs vs. 142 ± 18 in controls after three cycles (p<0.001).

**Table 3:** 16S sequencing and diversity statistics.

Parameter	Control	HFLF	5-ASA	p-Value	FDR
**Sequencing metrics**					

Raw reads (mean ± SD)	48.235 ± 5.123	51.342 ± 4.892	49.876 ± 5.234	0.342	–
Quality filtered reads	42.156 ± 4.234	45.234 ± 4.123	43.567 ± 4.567	0.287	–
ASVs detected	142 ± 18	287 ± 21	231 ± 19	<0.001	<0.001

**Alpha diversity (post-cycle 3)**					

Shannon index	2.1 ± 0.2	4.5 ± 0.2	3.8 ± 0.3	<0.001	<0.001
Chao1	156 ± 19	298 ± 23	245 ± 21	<0.001	<0.001
Faith’s PD	8.2 ± 1.1	15.6 ± 1.4	12.3 ± 1.2	<0.001	<0.001

**Beta diversity**					

PERMANOVA R^2^	–	0.42	–	<0.001	<0.001

Beta diversity analysis using PCoA of Bray-Curtis distances revealed distinct clustering by diet group (Figure 4D). PERMANOVA confirmed significant differences in community composition (R^2^=0.42, p<0.001), with diet explaining 42 % of variance and time/cycle explaining an additional 18 %. Weighted UniFrac analysis showed similar separation patterns, indicating phylogenetically distinct communities between diet groups.

### Differential microbial abundance

**Figure 5:**
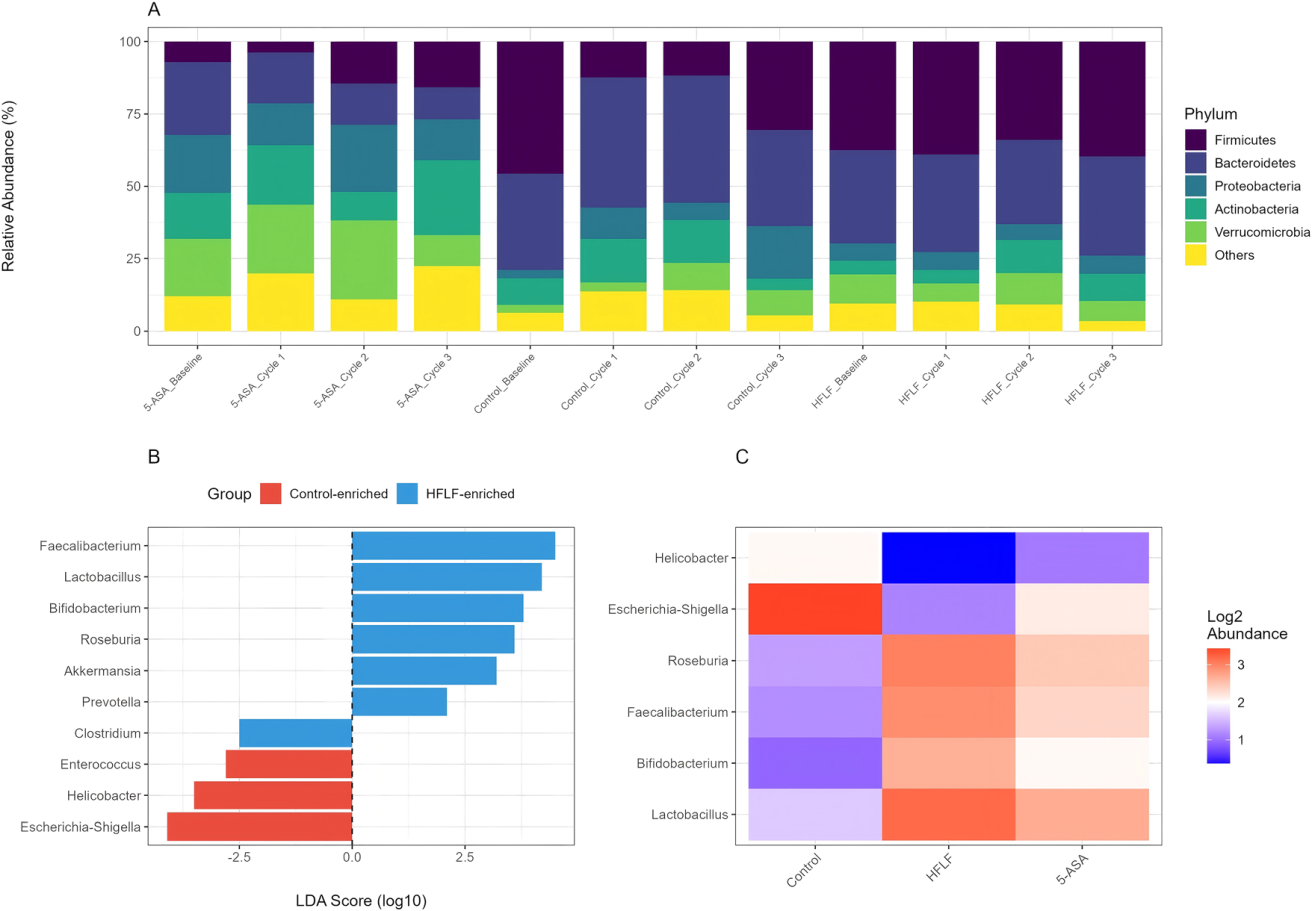
Differential microbial abundance analysis. (A) Phylum-level relative abundance across groups/timepoints. (B) LEfSe LDA score plot of differentially abundant genera between HFLF and control (|LDA|>2.0; FDR<0.05). (C) Heatmap of key genera abundance across groups (log2-scaled).

At the phylum level, HFLF diet prevented the dysbiotic shifts characteristic of DSS colitis (Figure 5A). Bacteroidetes, Firmicutes, Actinobacteria and Proteobacteria were the primary microbiota [14], 15]. Control mice exhibited decreased Firmicutes (32.4 ± 3.1 % vs. baseline 48.2 ± 2.8 %, p<0.01) and increased Proteobacteria (18.7 ± 2.3 % vs. baseline 3.2 ± 0.8 %, p<0.001). HFLF mice maintained Firmicutes at 44.1 ± 2.9 % and limited Proteobacteria expansion to 6.8 ± 1.2 % (p<0.001 vs. control).

LEfSe analysis identified 47 differentially abundant taxa between groups (LDA score >2.0) (Figure 5B and C). HFLF diet enriched beneficial genera including Lactobacillus (8.2 ± 1.1 % vs. 2.1 ± 0.4 % in controls, p<0.001), Bifidobacterium (5.3 ± 0.8 % vs. 0.9 ± 0.2 %, p<0.001), and Faecalibacterium (6.7 ± 0.9 % vs. 1.3 ± 0.3 %, p<0.001). A decrease of the butyrate-producing species *Roseburia hominis* and *Faecalibacterium prausnitzii* defines dysbiosis in patients with ulcerative colitis [16]. Our findings showed HFLF diet specifically promoted these butyrate producers, with Roseburia increasing 4.8-fold (p<0.001).

Potentially pathogenic taxa were suppressed by HFLF diet, including Escherichia-Shigella (1.2 ± 0.3 % vs. 9.8 ± 1.4 % in controls, p<0.001) and Helicobacter (0.3 ± 0.1 % vs. 3.2 ± 0.6 %, p<0.001). The Firmicutes/Bacteroidetes ratio, often altered in IBD, was preserved in HFLF mice (1.4 ± 0.2) vs. controls (0.6 ± 0.1, p<0.001).

### SCFA production and functional predictions

**Figure 6:**
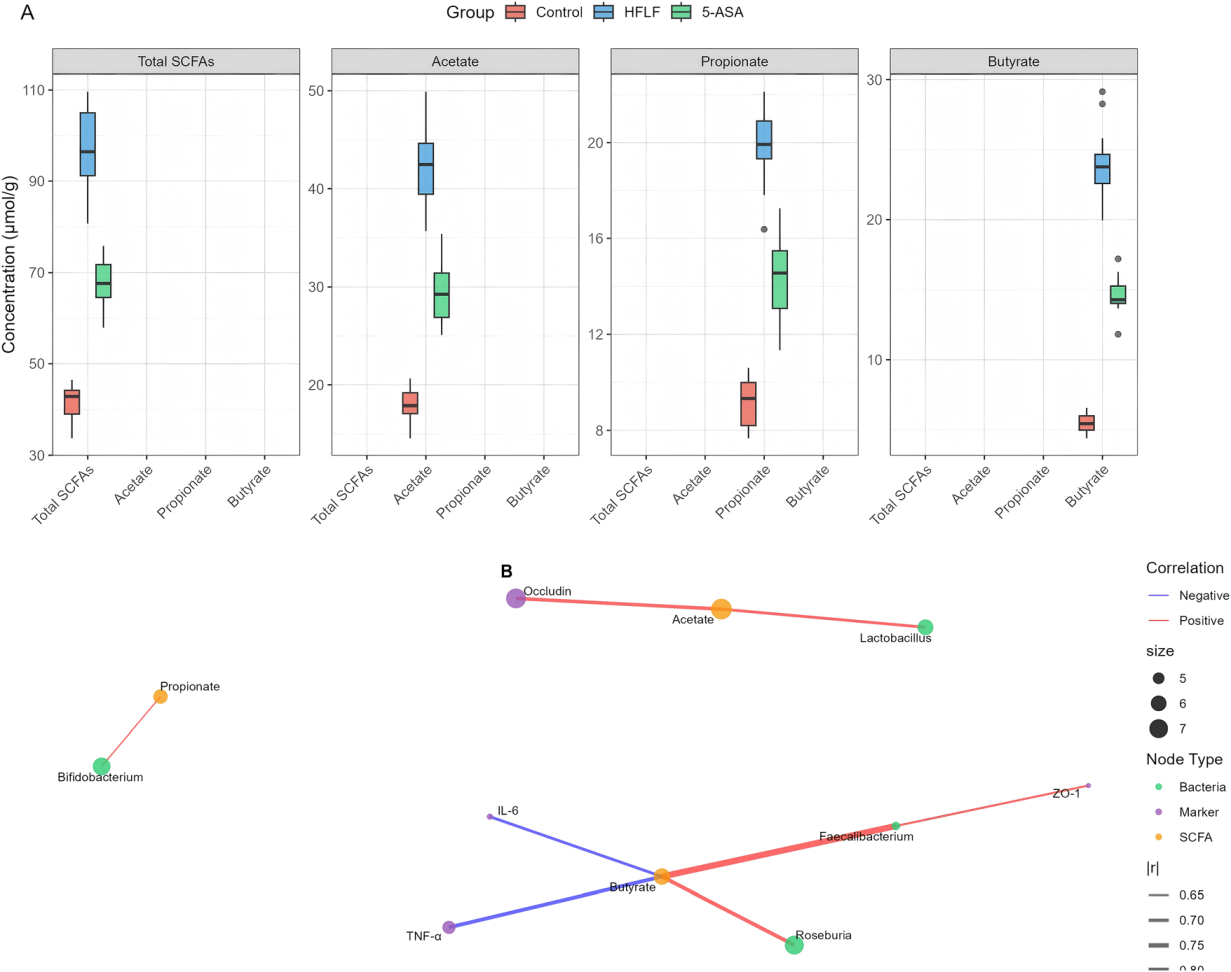
Metabolic profiles and correlation network. (A) Fecal SCFA concentrations (total, acetate, propionate, butyrate) by group; boxplots show median, IQR, and whiskers (1.5 × IQR). (B) Spearman correlation network linking bacterial genera, SCFAs, and inflammatory/barrier markers. Node color indicates type (bacteria/SCFA/marker); edge color red/blue=positive/negative; edge width ∝ |r|. Only associations with |r|≥0.5 and FDR<0.05 are displayed.

SCFA analysis revealed substantial metabolic shifts induced by HFLF diet (Figure 6A). Total SCFA concentration in feces increased from 42.3 ± 4.1 μmol/g in controls to 98.7 ± 7.2 μmol/g in HFLF mice (p<0.001). Individual SCFA changes included: acetate (18.2 ± 2.1–42.3 ± 3.8 μmol/g, p<0.001), propionate (8.7 ± 1.2–19.8 ± 2.1 μmol/g, p<0.001), and most notably, butyrate (6.1 ± 0.8–24.3 ± 2.7 μmol/g, p<0.001), representing a 4-fold increase. The butyrate: total SCFA ratio increased from 14.4 % in controls to 24.6 % in HFLF mice (p<0.001).

PICRUSt2 functional predictions identified enrichment of carbohydrate metabolism pathways in HFLF mice, including starch and sucrose metabolism (p<0.001), glycolysis/gluconeogenesis (p<0.01), and pyruvate metabolism (p<0.01). Pathways related to SCFA production, particularly butyryl-CoA:acetate CoA-transferase (K01034) and butyrate kinase (K00929), showed 2.3-fold and 1.9-fold increases respectively (p<0.001). Tax4Fun analysis provided corroborative evidence, confirming enrichment of genes involved in carbohydrate fermentation and SCFA biosynthesis pathways in HFLF-fed mice, with consistent patterns observed for acetyl-CoA synthesis and butyrate production enzymes.

### Correlation and integration analysis

Spearman correlation analysis revealed strong associations between microbial taxa, SCFAs, and host markers (Figure 6B, Table 4). Butyrate levels showed the strongest negative correlations with inflammatory markers (TNF-α: r=−0.72, p<0.001; IL-6: r=−0.68, p<0.001) and positive correlations with barrier proteins (ZO-1: r=0.81, p<0.001; Occludin: r=0.76, p<0.001). Faecalibacterium abundance correlated positively with butyrate production (r=0.83, p<0.001) and negatively with DAI scores (r=−0.71, p<0.001).

**Table 4:** Mixed-effects model results.

Parameter	Estimate (β ± SE)	t-value	p-Value	ΔAIC
**Disease activity index model**				

Intercept	10.23 ± 0.82	12.48	<0.001	–
Diet (HFLF)	−4.82 ± 0.76	−6.34	<0.001	−28.3
Time	0.18 ± 0.03	6.00	<0.001	−15.2
Diet × Time	−0.12 ± 0.02	−6.00	<0.001	−18.7
Random effects, SD	1.23	–	–	–

**Shannon diversity model**				

Intercept	3.12 ± 0.18	17.33	<0.001	–
Diet (HFLF)	2.13 ± 0.21	10.14	<0.001	−42.1
Cycle	−0.52 ± 0.08	−6.50	<0.001	−19.3
Diet × Cycle	0.31 ± 0.06	5.17	<0.001	−12.8
Random effects, SD	0.34	–	–	–

RDA analysis showed that diet and SCFA levels explained 58 % of variance in inflammatory marker expression (adjusted R^2^=0.58, p<0.001). Mediation analysis suggested that 43 % of HFLF diet’s anti-inflammatory effect was mediated through increased butyrate production (indirect effect: *β*=−0.42, 95 % CI: −0.58 to −0.26, p<0.001).

Mantel tests confirmed significant correlations between microbiota composition and inflammatory status (r=0.64, p<0.001), with stronger associations in HFLF mice (r=0.71) than controls (r=0.48), suggesting enhanced microbiota-host coupling under dietary intervention.

### Sensitivity and subgroup analyses

Sex-stratified analysis (conducted in a subset with both male and female mice) showed consistent HFLF effects across sexes, though females exhibited slightly stronger improvements in barrier function markers (interaction p=0.08). Initial body weight stratification revealed no significant interaction effects, indicating robust dietary benefits regardless of baseline weight. Varying DSS concentrations in pilot experiments (2.0, 2.5, 3.0 %) confirmed that HFLF protection was maintained across different disease severities, though effect sizes were greatest at 2.5 % DSS. The timing of dietary intervention initiation (tested at −14, −7, and 0 days relative to first DSS) showed optimal effects with 7-day pre-treatment, though benefits were still significant even when diet was started concurrently with DSS.

## Discussion

This study provides comprehensive evidence that HFLF dietary intervention exerts sustained protective effects throughout recurrent DSS-induced colitis cycles by orchestrating beneficial changes in gut microbiota composition, enhancing SCFA production, and modulating inflammatory and barrier functions. Our findings extend beyond previous acute colitis studies by demonstrating that dietary fiber’s benefits persist and potentially strengthen across multiple inflammatory challenges, offering important insights for long-term IBD management strategies.

The observation that HFLF diet progressively enhanced protection with successive DSS cycles represents a novel finding with significant implications. While preventive use of a high fiber diet protects mice against acute and chronic damage induced by DSS in mice [17], our results demonstrate that this protection not only persists but amplifies during recurrent inflammation. This progressive enhancement likely reflects cumulative microbiota remodeling and metabolic adaptation, as evidenced by the steadily increasing butyrate production and enrichment of beneficial taxa across cycles. The strengthening protection contrasts with the progressive deterioration observed in control mice, highlighting the divergent trajectories established by dietary intervention.

Our microbiota analyses revealed that HFLF diet prevented the characteristic dysbiosis associated with DSS colitis, maintaining diversity and compositional stability despite repeated inflammatory insults. The preservation of alpha diversity is particularly noteworthy given that mucosal microbial diversity is reduced in IBD, particularly in CD [18]. The enrichment of butyrate-producing bacteria, including Faecalibacterium and Roseburia, aligns with human IBD studies showing depletion of these taxa in active disease [19]. Importantly, we observed that HFLF diet not only preserved these beneficial bacteria but actually increased their abundance above baseline levels by the third cycle, suggesting an adaptive response that enhances colonization resistance against pathobionts.

The substantial increase in SCFA production, particularly the 4-fold elevation in butyrate levels, provides a mechanistic link between microbiota modulation and host protection. SCFAs, such as acetate, propionate and butyrate, are important metabolites in maintaining intestinal homeostasis [20]. Our correlation analyses strongly support a contributory role for SCFAs in mediating dietary benefits, with butyrate showing the strongest associations with both inflammatory suppression and barrier enhancement. The literature indicates that butyrate can suppress lipopolysaccharide (LPS)-induced NF-κB activation via GPR109A [21], which is consistent with our finding of reduced NF-κB signaling in HFLF mice. However, while our data suggest this mechanism may be operative in our model, we acknowledge that the precise molecular pathways were not directly investigated in the current study. Future mechanistic experiments employing GPR109A knockout mice or selective receptor antagonists would be valuable to definitively establish this pathway’s contribution to the observed protective effects. Furthermore, the preserved expression of tight junction proteins in HFLF mice corresponds with evidence that SCFAs have been shown to regulate intestinal permeability [22]. Previous studies have demonstrated that dietary supplementation with conjugated linoleic acid increased the epithelial expression of zonulin-1, occludin, and claudin-3, and ameliorated dextran sodium sulphate (DSS)-induced colitis in mice [14], supporting the concept that lipid-based interventions can modulate barrier function through similar mechanisms.

The complexity of dietary fiber effects in colitis deserves careful consideration. While our results demonstrate clear benefits of a mixed fiber approach, previous studies have shown that addition of inulin, but not the insoluble fiber cellulose, further exacerbated the severity of colitis [23]. This apparent contradiction highlights the importance of fiber type, dose, and dietary context. Our HFLF diet combined multiple fiber sources with complementary properties: cellulose for bulk, inulin for prebiotic effects, and pectin for gel formation and moderate fermentation. This combination may avoid the potential adverse effects of single, highly fermentable fibers while maximizing beneficial outcomes. The low-fat component of our diet likely contributed additional benefits by reducing pro-inflammatory lipid mediators and bile acid stress on the intestinal epithelium.

The sustained upregulation of barrier proteins, particularly ZO-1, occludin, and claudin-1, throughout recurrent inflammation represents a critical protective mechanism. A defective intestinal TJ barrier is an important contributing factor to the pathogenesis of various inflammatory conditions of the gut including inflammatory bowel disease [24]. Our findings that HFLF diet not only preserved but enhanced tight junction expression above baseline levels suggests active barrier reinforcement rather than mere protection from damage. The parallel increase in Muc2 expression indicates comprehensive barrier enhancement encompassing both epithelial and mucus layers.

The translational potential of our findings must be considered within the context of both opportunities and challenges. The 30 % total dietary fiber content employed in this study, while demonstrating robust efficacy in the murine model, represents a substantial increase over typical human dietary fiber intake (averaging 15–20 g/day in Western populations). Although this fiber level remains theoretically achievable through combinations of whole foods (such as legumes, whole grains, vegetables, and fruits) supplemented with purified fiber sources, several practical considerations merit careful examination. First, the gastrointestinal tolerance of such high fiber intake in IBD patients, particularly during active disease phases, remains uncertain. IBD patients frequently experience symptoms including bloating, abdominal discomfort, and altered bowel habits that may be exacerbated by rapid increases in dietary fiber. The fermentation of high fiber loads can produce excessive gas and osmotic effects that could potentially worsen symptoms in susceptible individuals. Second, the feasibility of maintaining this fiber intake level requires substantial dietary modification and patient adherence, which may be challenging in clinical practice. Third, individual variation in baseline microbiota composition and disease phenotype may influence response to high-fiber interventions, necessitating personalized approaches rather than universal recommendations. Our study utilized a gradual introduction of the HFLF diet with a 7-day pre-treatment period, which may have facilitated microbiota adaptation; however, patients presenting with active disease may not tolerate such fiber levels initially. Therefore, while our findings provide strong preclinical evidence for the therapeutic potential of HFLF dietary intervention, clinical translation will likely require carefully designed dose-escalation protocols, close monitoring of patient tolerance, and potentially individualized fiber prescriptions based on disease activity, microbiome status, and symptom response. Future human studies should investigate optimal fiber dosing strategies that balance therapeutic efficacy with practical tolerability and long-term adherence.

The progressive nature of protection observed suggests that even partial dietary improvements might yield cumulative benefits over time in chronic inflammatory conditions. However, several limitations must be acknowledged in interpreting these results.

First, while the DSS model effectively mimics certain IBD features, it does not fully recapitulate the complex immune dysregulation characteristic of human disease. The primarily chemical-induced injury may not reflect the T cell-mediated pathogenesis prominent in Crohn’s disease or the autoimmune components of ulcerative colitis. Second, our 16S rRNA sequencing approach, while informative for community profiling, lacks the resolution to identify strain-level variations and functional genes that might be critical for therapeutic effects. Future studies employing metagenomic and metatranscriptomic approaches could provide deeper mechanistic insights into the functional capacity of the modulated microbiota.

The timing and duration of dietary intervention represent important considerations for clinical translation. Our protocol initiated dietary changes one week before the first DSS challenge, allowing microbiota adaptation before inflammatory stress. In clinical practice, patients often seek dietary interventions after disease onset, and our sensitivity analysis showing benefits even with concurrent diet initiation provides encouraging evidence for therapeutic applications. Nevertheless, the optimal timing for maximizing therapeutic benefits while minimizing potential adverse effects during active inflammation requires further investigation.

The mechanisms underlying progressive protection across cycles warrant additional exploration. Potential explanations include cumulative expansion of beneficial bacterial populations, enhanced epithelial adaptation to fiber-derived metabolites, and epigenetic modifications induced by sustained SCFA exposure. The observation that certain beneficial effects, particularly barrier protein expression, exceeded baseline levels by the third cycle suggests active remodeling rather than simple preservation. Investigation of epithelial stem cell dynamics, tissue-resident memory responses, and chromatin accessibility changes could elucidate these adaptive mechanisms.

Future research directions should include comparative evaluation of different fiber combinations to optimize therapeutic effects while minimizing potential adverse responses. Given the heterogeneity of IBD presentations and the emerging concept of personalized nutrition, studies examining how baseline microbiota composition influences dietary responsiveness could guide patient stratification strategies. Additionally, investigation of synergistic combinations with other therapeutic modalities, such as biologics or small molecule inhibitors, could enhance treatment outcomes.

The potential for HFLF dietary intervention to serve as a preventive strategy in high-risk populations deserves exploration. Our findings of cumulative protection suggest that long-term dietary modification might reduce disease incidence or severity in genetically susceptible individuals. Prospective cohort studies examining dietary patterns and IBD development, coupled with mechanistic studies in genetically engineered mouse models, could establish preventive dietary guidelines.

## Conclusions

This study demonstrates that HFLF dietary intervention provides sustained and progressive protection against recurrent DSS-induced colitis through coordinated modulation of the gut microbiota-SCFA-inflammation axis. The preservation of microbial diversity, enrichment of beneficial taxa, enhanced SCFA production, and reinforcement of intestinal barrier function collectively contribute to improved outcomes across multiple inflammatory cycles. These findings support the therapeutic potential of dietary fiber intervention as an adjunctive strategy in IBD management and highlight the importance of sustained dietary modification for optimal benefits. Future clinical trials translating these findings to human IBD populations are warranted to establish evidence-based dietary recommendations for this challenging chronic inflammatory condition.

## References

[1] Sepanlou SG, Ikuta K, Vahedi H, Bisignano C, Safiri S, GBD 2017 Inflammatory Bowel Disease Collaborators (2020). The global, regional, and national burden of inflammatory bowel disease in 195 countries and territories, 1990–2017: a systematic analysis for the Global Burden of Disease Study 2017. Lancet Gastroenterol Hepatol.

[2] Liverani E, Scaioli E, Digby RJ, Bellanova M, Belluzzi A (2016). How to predict clinical relapse in inflammatory bowel disease patients. World J Gastroenterol.

[3] Glassner KL, Abraham BP, Quigley EMM (2020). The microbiome and inflammatory bowel disease. J Allergy Clin Immunol.

[4] Ventura I, Chomon-García M, Tomás-Aguirre F, Palau-Ferré A, Legidos-García ME, Murillo-Llorente MT (2024). Therapeutic and immunologic effects of short-chain fatty acids in inflammatory bowel disease: a systematic review. Int J Mol Sci.

[5] Armstrong H, Mander I, Zhang Z, Armstrong D, Wine E (2021). Not all fibers are born equal; variable response to dietary fiber subtypes in IBD. Front Pediatr.

[6] Silveira ALM, Ferreira AVM, de Oliveira MC, Rachid MA, da Cunha Sousa LF, Dos Santos Martins F (2017). Preventive rather than therapeutic treatment with high fiber diet attenuates clinical and inflammatory markers of acute and chronic DSS-induced colitis in mice. Eur J Nutr.

[7] Parada Venegas D, De la Fuente MK, Landskron G, González MJ, Quera R, Dijkstra G (2019). Short chain fatty acids (SCFAs)-mediated gut epithelial and immune regulation and its relevance for inflammatory bowel diseases. Front Immunol.

[8] Wark G, Samocha-Bonet D, Ghaly S, Danta M (2020). The role of diet in the pathogenesis and management of inflammatory bowel disease: a review. Nutrients.

[9] Shin Y, Han S, Kwon J, Ju S, Choi TG, Kang I (2023). Roles of short-chain fatty acids in inflammatory bowel disease. Nutrients.

[10] Miles JP, Zou J, Vijay-Kumar MV, Pellizzon M, Ulman E, Ricci M (2017). Supplementation of low- and high-fat diets with fermentable fiber exacerbates severity of DSS-induced acute colitis. Inflamm Bowel Dis.

[11] Chassaing B, Aitken JD, Malleshappa M, Vijay-Kumar M (2014). Dextran sulfate sodium (DSS)-induced colitis in mice. Curr Protoc Im.

[12] Nakase H, Uchino M, Shinzaki S, Matsuura M, Matsuoka K, Kobayashi T (2021). Evidence-based clinical practice guidelines for inflammatory bowel disease 2020. J Gastroenterol.

[13] Gerasimidis K, Godny L, Sigall-Boneh R, Svolos V, Wall C, Halmos E (2021). Current recommendations on the role of diet in the aetiology and management of IBD. Frontline Gastroenterol.

[14] Chen Y, Yang B, Ross RP, Jin Y, Stanton C, Zhao J (2019). Orally administered CLA ameliorates DSS-induced colitis in mice via intestinal barrier improvement, oxidative stress reduction, and inflammatory cytokine and gut microbiota modulation. J Agric Food Chem.

[15] Ferenc K, Jarmakiewicz-Czaja S, Filip R (2022). Components of the fiber diet in the prevention and treatment of IBD-an update. Nutrients.

[16] Machiels K, Joossens M, Sabino J, De Preter V, Arijs I, Eeckhaut V (2014). A decrease of the butyrate-producing species Roseburia hominis and Faecalibacterium prausnitzii defines dysbiosis in patients with ulcerative colitis. Gut.

[17] Nagy-Szakal D, Hollister EB, Luna RA, Szigeti R, Tatevian N, Smith CW (2013). Cellulose supplementation early in life ameliorates colitis in adult mice. PLoS One.

[18] Wang R, Li Z, Liu S, Zhang D (2023). Global, regional and national burden of inflammatory bowel disease in 204 countries and territories from 1990 to 2019: a systematic analysis based on the Global Burden of Disease Study 2019. BMJ Open.

[19] Frank DN, Robertson CE, Hamm CM, Kpadeh Z, Zhang T, Chen H (2011). Disease phenotype and genotype are associated with shifts in intestinal-associated microbiota in inflammatory bowel diseases. Inflamm Bowel Dis.

[20] Parada Venegas D, De la Fuente MK, Landskron G, González MJ, Quera R, Dijkstra G (2019). Short chain fatty acids (SCFAs)-mediated gut epithelial and immune regulation and its relevance for inflammatory bowel diseases. Front Immunol.

[21] Canani RB, Costanzo MD, Leone L, Pedata M, Meli R, Calignano A (2011). Potential beneficial effects of butyrate in intestinal and extraintestinal diseases. World J Gastroenterol.

[22] Pérez-Reytor D, Puebla C, Karahanian E, García K (2021). Use of short-chain fatty acids for the recovery of the intestinal epithelial barrier affected by bacterial toxins. Front Physiol.

[23] Miles JP, Zou J, Kumar MV, Pellizzon M, Ulman E, Ricci M (2017). Supplementation of low- and high-fat diets with fermentable fiber exacerbates severity of DSS-induced acute colitis. Inflamm Bowel Dis.

[24] Turner JR (2009). Intestinal mucosal barrier function in health and disease. Nat Rev Immunol.

